# Zirconia/Hydroxyapatite Composites Synthesized Via Sol-Gel: Influence of Hydroxyapatite Content and Heating on Their Biological Properties

**DOI:** 10.3390/ma10070757

**Published:** 2017-07-05

**Authors:** Flavia Bollino, Emilia Armenia, Elisabetta Tranquillo

**Affiliations:** 1 Department of Industrial and Information Engineering, University of Campania “Luigi Vanvitelli”, 81031 Aversa, Italy; elisabetta.tranquillo@unicampania.it; 2 Department of Cardiothoracic and Respiratory Sciences, University of Campania “Luigi Vanvitelli”, 80131 Naples, Italy; emiliaarmenia@hotmail.it

**Keywords:** sol-gel method, Fourier transform infrared spectroscopy (FTIR) analysis, bioactivity, biocompatibility

## Abstract

Zirconia (ZrO_2_) and zirconia-based glasses and ceramics are materials proposed for use in the dental and orthopedic fields. In this work, ZrO_2_ glass was modified by adding different amounts of bioactive and biocompatible hydroxyapatite (HAp). ZrO_2_/HAp composites were synthesized via the sol-gel method and heated to different temperatures to induce modifications of their chemical structure, as ascertained by Fourier transform infrared spectroscopy (FTIR) analysis. The aim was to investigate the effect of both HAp content and heating on the biological performances of ZrO_2_. The materials’ bioactivity was studied by soaking samples in a simulated body fluid (SBF). FTIR and scanning electron microscopy (SEM)) analyses carried out after exposure to SBF showed that all materials are bioactive, i.e., they are able to form a hydroxyapatite layer on their surface. Moreover, the samples were soaked in a solution containing bovine serum albumin (BSA). FTIR analysis proved that the synthesized materials are able to adsorb the blood protein, the first step of cell adhesion. WST-8 ([2-(2-methoxy-4-nitrophenyl)-3-(4-nitrophenyl)-5-(2,4-disulfophenyl)-2H-tetrazolium, monosodium salt]) assay showed that no cytotoxicity effects were induced by the materials’ extract. However, the results proved that bioactivity increases with both the HAp content and the temperature used for the thermal treatment, whereas biocompatibility increases with heating but is not affected by the HAp content.

## 1. Introduction

Zirconia and zirconia-based glasses and ceramics have attracted considerable interest as materials to be used in the biomedical field [1,2,3,4,5]. In vivo and in vitro studies showed that zirconia and zirconia-based glasses and ceramics do not induce any cytotoxicity effects in either soft or hard tissue [3,5]. When a zirconia prosthesis was implanted in vivo, it was encapsulated by connective tissue, and thus any local or systemic toxic effect was not recorded after its insertion [3,5]. However, although zirconia is well tolerated, it is a bioinert material, as it does not show either the ability of direct bone bonding or osteoconduction behavior. Moreover, release of residues or degradation phenomena of zirconia implants were not detected and a low bacterial growth was observed [3,5]. In addition to these biological properties, the good mechanical behavior of zirconia has stimulated the interest of researchers in the biomaterials field. ZrO_2_ is an oxide which presents three types of crystalline structures at ambient pressure: (i) the monoclinic phase (m-ZrO_2_), which is stable from room temperature up to 1170 °C and exhibits poor mechanical properties; (ii) the tetragonal phase (t-ZrO_2_), which is stable in the temperature range 1170–2370 °C and has good mechanical properties; and (iii) the cubic phase (c-ZrO_2_), which is stable above 2370 °C and has moderate mechanical properties [6,7,8]. In order to stabilize the tetragonal phase at room temperature, zirconia can be mixed with other metallic oxides (e.g., MgO, La_2_O_3_, CaO, Y_2_O_3_) to obtain strong ceramics. Among such materials, yttrium-stabilized zirconia (known as tetragonal zirconia polycrystal (TZP)) is one of the most studied [9]. As their mechanical properties, such as resistance to traction and compression resistance, are similar to those of metals (e.g., stainless steel) [10], and they have good biocompatibility, zirconia and zirconia-based ceramics were first used in orthopedics as a substitute for titanium and alumina in hip head prostheses [11]. Afterwards, they were successfully used in dentistry, where they are still considered to be a material of choice for root canal posts, fixed partial dentures, and dental implants [12]. Moreover, some studies [13,14,15,16] report the use of TZP as fillers to reinforce the mechanical properties of synthetic hydroxyapatite (HAp) (a biodegradable and biocompatible calcium phosphate ceramic with a bone-like structure, capable of forming strong chemical bonds with natural bone tissue [17]). Other studies investigated the use of zirconia-based glasses, synthesized via the sol-gel method, as drug delivery systems [2,18] or as coatings capable of improving the biological performance of titanium implants [19,20,21,22]. Zirconia-based glasses, synthesized by means of the sol-gel method, showed the ability to slightly improve cell viability of the human osteosarcoma cell line (SAOS-2) [20] and human mesenchymal stromal cells (hMSCs) [22]. This is ascribable mainly to the preparation method. The sol-gel technique, indeed, is a versatile process used to make glass and ceramic materials at low temperature, and has been extensively used to prepare a wide variety of materials with different applications, including the bioglass [23,24,25,26,27]. The process starts from a solution of metal alkoxide or metal salt precursors in water-alcohol and involves their hydrolysis and condensation reactions, which lead to the formation of a 3D rigid gel [28]. By drying the obtained wet gel, it is possible to prepare xerogels (by exposure to low temperatures), aerogels (by solvent extraction under supercritical conditions), or dense ceramics and glasses by means of a further heat treatment at higher temperatures. Glasses and ceramics synthesized via the sol–gel method exhibit higher bioactivity and biocompatibility than materials with the same composition but prepared by melt–quenching [29,30,31]. Sol-gel-derived glasses have an inherent mesoporosity that gives them a larger surface area and potentially more rapid degradation rates than melt-derived glasses of a similar composition. Moreover, the presence of –OH groups on their surface stimulates hydroxyapatite nucleation, promoting easier osseointegration.

The aim of the present study has been to synthesize zirconia-based composites containing different amounts of HAp (xZrO_2_ (1 − x)HAp, with x = no stabilized ZrO_2_ mole fraction) via the sol-gel method in order to investigate the influence of the biocompatible and bioactive HAp on the biological response of the synthesized composites. Moreover, it is known [32,33] that both sol-gel zirconia and HAp glasses can crystallize by heating, leading to different crystalline phases. Therefore, the gel materials were heated at 120 °C, 600 °C, and 1000 °C to induce phase transformation and, in turn, to study the effect of thermal treatment on the biological response of the obtained materials. The choice of the temperature has been based on published studies [32,33]. Fourier transform infrared spectroscopy (FTIR) analysis was carried out to follow the materials’ structural modification, induced by both HAp addition and heating. Moreover, bioactivity and biocompatibility of all samples were studied by in vitro preliminary tests after the different thermal treatments.

## 2. Results and Discussion

### 2.1. Chemical Characterization

The heat treatments of ZrO_2_ and xZrO_2_·(1 − x)HAp gels led to transformations visible to the naked eye. The samples heated to 120 °C are yellow. After heating to 600 °C they became black, whereas after heating to 1000 °C the powders became white, according to literature [34,35]. The change in the sample color is ascribable to the transformation of the ZrO_2_ structure induced by heating and studied by [35,36] X-ray diffraction (XRD) and thermal analyses of zirconia, as reported in the literature [32,35,36,37]. It was shown that Acetil Acetone (AcAc)-containing zirconia, synthesized via the sol-gel method, is an amorphous and yellow-brown material, because a complex between the zirconium and the AcAc is formed in the sol phase, which exhibits strong absorption in the UV region and low-intensity absorption in the visible spectral region [38].

The increase in temperature leads to the condensation of surface –OH groups with the formation of H_2_O. The sites involved in this reaction are also the sites for the promotion of the nucleus formation of the tetragonal phase. At about 400 °C, the tetragonal phase begins to crystallize [32,37] and the material gradually darkens [34,35]. At 600 °C the material is entirely converted into the tetragonal phase (t-ZrO_2_) and it appears completely black [34,35]. This phenomenon is correlated to the formation of defects in the material, according to Wachsmanet al. [35]. The dihydroxylation process, which takes place on the material’s surface above 100 °C, leads to oxygen surface desorption and, thus, to the formation of oxygen vacancies [32]. The gradual transformation of t-ZrO_2_ into the monoclinic phase (m-ZrO_2_) occurs [32,37] when the temperature is further increased. Therefore, when the material was heat treated at 1000 °C, it was transformed completely into the monoclinic structure and appears white, indicating a lack of oxygen vacancies, as reported by Wachsman et al. [35].

**Figure 1:**
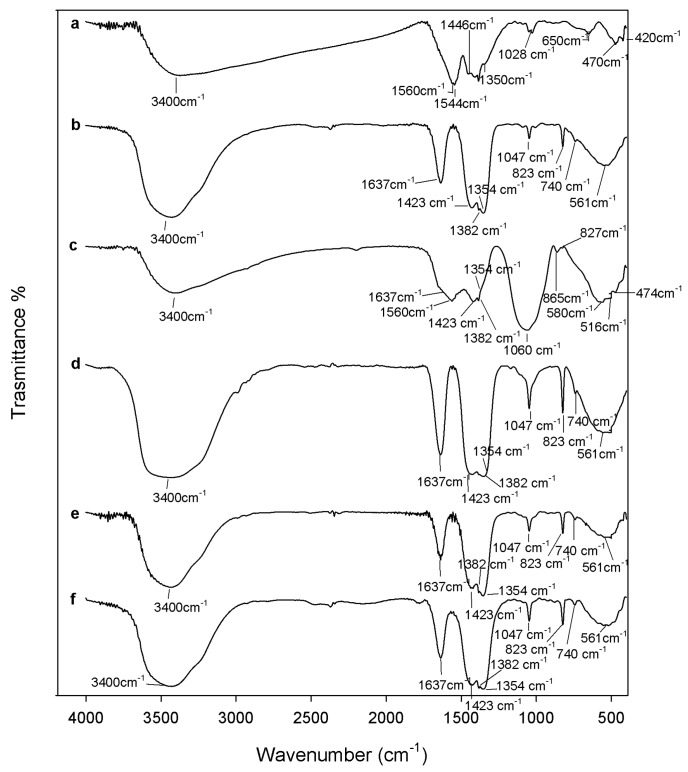
Fourier transform infrared spectroscopy (FTIR) of (**a**) ZrO_2_; (**b**) hydroxyapatite (Hap); (**c**) 9ZrO_2_∙1Hap; (**d**) 7ZrO_2_∙3Hap; and (**e**) 5ZrO_2_∙5HAp gels, heated to 120 °C and (**f**) Ca(NO_3_)_2_ 4H_2_O.

**Figure 2:**
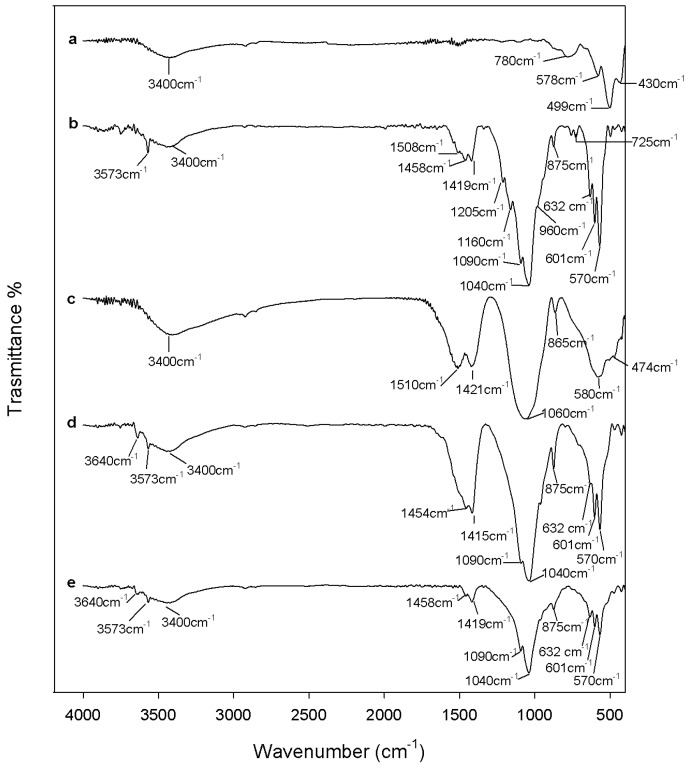
FTIR of (**a**) ZrO_2_; (**b**) Hap; (**c**) 9ZrO_2_∙1Hap; (**d**) 7ZrO_2_∙3Hap; and (**e**) 5ZrO_2_∙5HAp gels, heated to 600 °C.

**Figure 3:**
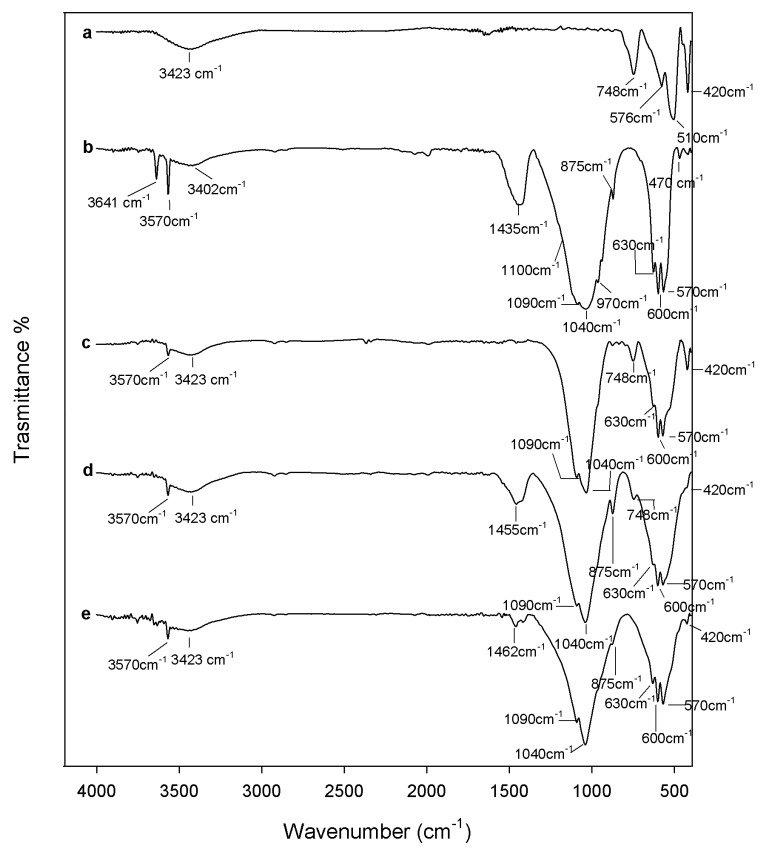
FTIR of (**a**) ZrO_2_; (**b**) Hap; (**c**) 9ZrO_2_∙1Hap; (**d**) 7ZrO_2_∙3Hap; and (**e**) 5ZrO_2_∙5HAp gels, heated to 1000 °C.

Despite the fact that HAp appears as a white powder regardless of the temperature used for heat treatment, the xZrO_2_·(1 − x)HAp composites retain the zirconia color variations. To follow the material transformations induced by heating, FTIR analysis of all samples was carried out as a function of the temperature (Figure 1, Figure 2 and Figure 3).

Figure 1 shows the FTIR spectra of all samples after drying at 120 °C. The FTIR spectrum of ZrO_2_ after 120 °C heating (Figure 2a) shows all peaks typical of amorphous acetyl acetone (AcAc) containing zirconia sol-gel materials [36,39]. The broad intense band at 3400 cm^−1^ is due to the vibrations of –OH groups in both physically bonded water and Zr-OH groups in the matrix. Moreover, the bands observed at 1560 cm^−1^ and 1446 cm^−1^ are assigned to C=O vibrations of AcAc bidentate binding. The bands at 1544 cm^−1^ and 1350 cm^−1^ are due to C–C vibrations. The peaks at 1028 cm^−1^ are assigned to C–C–H bending, mixed with stretching C–C vibrations of AcAc [39]. The bands at 650 cm^−1^ and 470 cm^−1^ are due to Zr–OH and Zr–O–Zr stretching, respectively [40,41], whereas the peak at 420 cm^−1^ is attributed to Zr–OAcAc vibrations [39].

The spectrum of HAp after 120 °C heating (Figure 1b) is very similar to the calcium nitrate spectrum (Figure 1f). Only the bands of the –OH stretching and banding (at 3400 cm^−1^ and 1637 cm^−1^ respectively), in the adsorbed water and of nitrate vibrations are visible, such as the signals related to asymmetric and symmetric stretching of nitrate ions at 1423, 1382, and 1354 cm^−1^ [42], the sharp peaks at 1047, 823 cm^−1^, and the weak peak at 738 cm^−1^, ascribable to the bending modes of the nitrate ions [42]. Calcium nitrate, indeed, was soluble in the sol and it remained in solution as the particles formed and coalesced. During drying at 120 °C, calcium nitrate coats the formed particles, therefore only the signals of the salt are visible, while those of the phosphate are masked. Only after the thermal degradation of nitrate ions (at temperatures over 500 °C [30]) does the calcium enter the network by diffusion, and the nitrate by-products are driven off. Therefore, a cleavage of the bridging oxygen bonds and the formation of non-oxygen bonds (and thus, ionic-crosslinkage with Ca^2+^ ions) occurs in the glass network [30,43,44]. Also, Catauro et al. [45] observed a similar phenomenon in SiO_2_-CaO-P_2_O_5_ ternary systems synthesized via sol-gel, where calcium nitrate was used as precursor of the CaO phase. The FTIR spectra of the materials dried at 120 °C were dominated by nitrate signals. However, the authors proved the presence of the silica and phosphate phases by FTIR analysis of the materials after soaking in a water solution. Only the signals of the silica and phosphate phases, and no signals of nitrates, were visible in the spectra. Moreover, IC analysis of the water solution showed that nitrate ions were released.

For the same reason the spectra of 0.7ZrO_2_·0.3HAp and 0.5ZrO_2_·0.5HAp samples (Figure 1d,e) are also very similar to the Ca(NO_3_)_2_·4H_2_O spectrum, whereas the spectrum of the 0.9ZrO_2_·0.1HAp (Figure 1c) sample also shows signals, due to ZrO_2_ and the phosphate phase. In particular, two intense bands are still visible in the region 1700–1300 cm^−1^, but with some differences in the shape and position compared to the ZrO_2_ spectrum. This can be due to the influence of: (i) nitrate vibrations in the region 1500–1300 cm^−1^, which also cause the appearance of the weak peak at 827 cm^−1^ [42]; and (ii) vibrations of carbonate ions not incorporated in the apatitic structure, in the region 1500–1400 cm^−1^ [46]. The presence of carbonate ions, due to the solubilization of atmospheric CO_2_ in the sol, is also proved by the presence of the weak peak at 865 cm^−1^ [14,45,46]. Moreover, (iii) it can be hypothesized that a new complex between calcium ions and AcAc in the sol phase is formed, and originates IR bands in the same region. It has been reported in literature [47], indeed, that calcium bis(acetylacetonate) is synthesized by adding calcium nitrate tetrahydrate and AcAc to an aqueous solution of ammonium hydroxide, which are reagents present in the sol of the xZrO_2_·(1 − x)HAp composites. Moreover, the intense band at 1060 cm^−1^ (ascribable to PO_4_^3−^ vibration [13,45,48,49]) and the broad band in the region 750–400 cm^−1^ suggest that an amorphous calcium phosphate was formed [46,50]. The presence of the PO_4_^3−^ vibration suggests that in this material the calcium nitrate does not coat the glass network. It can be explained by the formation of the complex between calcium ions and AcAc, which subtracts the Ca^2+^ ions by the solution, reducing the interaction between nitrate ions and the glass network. As in the 0.9ZrO_2_·0.1HAp sample, a lower ratio between the amount of HAp and ZrO_2_ is present; a higher ratio AcAc/Ca(NO_3_)_2_∙4H_2_O was present in the sol of this sample, compared to the other samples. Therefore, a higher amount of the Ca-AcAc complex was formed.

Figure 2 shows FTIR spectra of all samples after heating at 600 °C. In the FTIR spectrum of ZrO_2_ (Figure 2a) the peaks related to AcAc disappear due to its degradation [51], while sharp peaks in the region (generally assigned to the vibrations of Zr-O-Zr and Zr-OH bonds in a crystalline structure) appear [52,53], such as those at 780, 578, 499, and 430 cm^−1^. 

After 600 °C heating, degradation of the nitrate ions occurs and calcium ions enter into the network by diffusion [30]. Therefore, the spectra of HAp, of 0.7ZrO_2_·0.3Hap, and of 0.5ZrO_2_·0.5HAp composites (Figure 2b,d,e) completely change shape. In particular, FTIR analysis confirms that pure HAp was obtained (Figure 2b). All hydroxyapatite typical peaks, indeed, are visible. The intense bands at 1090 and 1040 cm^−1^, as well as the shoulder at 960 cm^−1^, are due to asymmetric and symmetric stretching in PO_4_^3−^ ions, respectively. The doublet at 601 and 570 cm^−1^ is due to phosphate bending modes and the sharp peaks at 3573 and 632 cm^−1^ are assigned to stretching and wagging vibrations of –OH groups in the crystalline apatite structure [14,54]. However, a calcium-deficient hydroxyapatite was obtained as proven by the signals of CO_3_^2−^ and HPO_4_^2−^ ions. In particular, the broad band in the range 1500–1400 cm^−1^ and the weak peak at 875 cm^−1^ are assigned to stretching and bending in the CO_3_^2−^ ions, which can substitute OH^−^ (type A substitution) or PO_4_^3−^ ions (more commune type B substitution) [54]. The signal at 875 cm^−1^ can also be ascribed to the presence of HPO_4_^2−^ that characterizes the non-stoichiometric HAp and indicates the formation of anhydrous dicalcium phosphate (DCPA, Ca_2_HPO_4_) [54,55]. As the two signals overlap, it is generally difficult to distinguish between CO_3_^2−^ and HPO_4_^2−^ groups. However, in the recorded HAp spectrum, the sharp peaks at 1205 cm^−1^, 1160 cm^−1^, and 725 cm^−1^ confirm the formation of DCPA. Those peaks, indeed, are due to the P-O vibrations in pyrophosphate ions, which originate by the condensation of two HPO_4_^2−^ to P_2_O_7_^4−^ [55,56]. The spectra of 0.7ZrO_2_·0.3HAp and 0.5ZrO_2_·0.5HAp composites (Figure 2d,e) show the typical signals of carbonated HAp, but the carbonate bands have a different shape and intensity. The shape of this band mainly depends on the presence of “non-apatitic” or “apatitic” CO_3_^2−^ ions, and in the latter case, on the substitution type in the HAp lattice (A or B type) [46]. The complexity of this band in the HAp spectrum (Figure 2b) suggests that different types of CO_3_^2−^ ions are present. Generally, a doublet shape in the region 1450–1410 cm^−1^, coupled to a weak peak at 870–875 cm^−1^, is due to the vibrations of type B carbonate, whereas in the region 1450–1550 cm^−1^ vibrations of type A carbonate are visible, which generally are coupled with a band at 880 cm^−1^ [57,58]. However, at 1500 and 1420 cm^−1^, vibrations of non-apatitic CO_3_^2−^ ions are also present, coupled with a weak peak at about 866 cm^−1^ [58]. Therefore, in HAp samples, where peaks at 1530 cm^−1^, 1455 cm^−1^, and 1415 cm^−1^ are distinguishable, apatitic type A and B carbonate ions are also present [59]. In the spectra of 0.7ZrO_2_·0.3HAp and 0.5ZrO_2_·0.5HAp composites (Figure 2d,e), the typical doublet of apatitic CO_3_^2^ type B is visible at 1455 cm^−1^ and 1415 cm^−1^ [57]. It cannot be excluded that the vibrations of CaCO_3_, produced by the thermal decomposition of calcium acetylacetonate [47], contribute to the increase of the doublet intensity, visible by comparing the HAp spectrum to the composite spectra and the composite spectra to each other. Its production and, thus, the band intensity, grows with zirconia content in the materials, due to the higher amount of AcAc used in the synthesis. The presence of the sharp peak at 3640 cm^−1^, ascribable to –OH stretching in calcium hydroxide, suggests that the formation of a CaO phase [50] also occurred in the 0.7ZrO_2_·0.3HAp samples. The spectrum of the 0.9ZrO_2_·0.1HAp sample (Figure 2c) shows that the formation of the HAp structure still had not occurred. This can be due to the high amount of zirconia. Other works [60,61] in literature reported the reduction of the HAp crystallinity extent, due to the incorporation of silica or zirconia in the HAp lattice. Therefore, not many differences are evident by comparing this spectrum to the spectra of the 120 °C heated samples (Figure 1c), except for the position and intensity of the doublet in the region 1500–1300 cm^−1^. The signals are shifted to 1510 cm^−1^ and 1420 cm^−1^, and the weak peak at 865 cm^−1^ is still present in the spectrum. This suggests the prevalence of non-apatitic carbonate ions. Moreover, its higher intensity compared to pure Hap, 0.7ZrO_2_·0.3HAp and 0.5ZrO_2_·0.5HAp composites can be ascribed to the degradation of the higher calcium acetylacetonate content, due to the use of the higher amount of AcAc in the synthesis. 

Figure 3 shows the FTIR spectra of all samples after 1000 °C heating. All peaks in the spectrum of the ZrO_2_ sample (Figure 3a) appear more intense and sharp compared to the spectrum of ZrO_2_ after 600 °C heating (Figure 2a). This observation suggests that an increase of sample crystallinity occurred. Moreover, the broad weak band at 718 cm^−1^ was replaced by an intense peak at 748 cm^−1^, typical of the m-ZrO_2_ spectrum [52].

After 1000° heating of the pure HAp sample the degradation of DCPA occurred, as proved by the disappearance of P_2_O_7_^4−^ peaks from the spectrum (Figure 3b) [54,55,56]. It is known [62] that calcium-deficient hydroxyapatite contains hydrogen phosphate ions which condense to pyrophosphate in the temperature range 600–700 °C. The pyrophosphate formed, in turn, degrades at higher temperatures producing HAp and tricalcium phosphate polymorphs β (β-TCP). The formation of β-TCP as a secondary phase is confirmed by the shoulders at 970 and 1100 cm^−1^ [54]. Moreover, the carbonate peaks are still present after 1000 °C heating, but with different shapes. Part of the CaCO_3_, indeed, decomposes into CO_2_ and CaO. The first is released as a volatile gas; the second is retained in the material, as proved by the sharp peak at 3641 cm^−1^ [50,63]. Therefore, the single band at 1435 cm^−1^ and the weak peak at 875 cm^−1^ are ascribable to the residual apatitic B type carbonate. After 1000 °C heating, the formation of HAp also occurred in the 0.9ZrO_2_·0.1HAp sample (Figure 3c), as proven by the modification of the FTIR spectrum, which assumes the typical shape of the HAp spectrum. The broad band in the region 700–400 cm^−1^ and the strong peak at 1060 cm^−1^ split, and the typical HAp doublets arise at 600–570 cm^−1^ and 1090–1040 cm^−1^, respectively. Moreover, the signal of the –OH groups in HAp are also visible at 3570 and 630 cm^−1^, whereas the carbonate signals disappear, confirming that such ions were not strictly incorporated in the materials structure (non apatitic carbonate). However, the peaks at 748 and 420 cm^−1^, and the shoulder at 510 cm^−1^, prove the presence of ZrO_2_ in the sample. ZrO_2_ peaks are still visible in the spectrum of the 1000° heated 0.7ZrO_2_·0.3HAp sample (Figure 3d), but with lower intensity and as shoulders, whereas they are not detectable in the spectra of the 0.5ZrO_2_·0.5HAp sample (Figure 3e) because they are overlapped by HAp signals. In the spectra of the 1000° heated 0.7ZrO_2_·0.3HAp and 0.5ZrO_2_·0.5HAp samples, the signal of CO_3_^2−^ type B residues are also visible. The band at 1455 cm^−1^ and the weak peak of 875 cm^−1^, indeed, are ascribable to residual apatitic carbonate [46,50]. Moreover, the formation of β-TCP, as a degradation product of the thermal degradation of the HAp phases, probably occurs also in those composites samples. In the literature, indeed, it is reported that zirconia can act as a catalyst of the decomposition reaction [61], which, thus, can also occur at a lower temperature [13]. However, β-TCP is undetectable in the spectra of the composites, because in those materials a lower HAp content is present which, thus, leads to the formation of a lower β-TCP amount.

### 2.2. Evaluation of Biological Properties

The biocompatibility of the synthesized samples after 600 °C and 1000 °C heating was assessed by evaluating both the materials’ ability to absorb blood proteins on their surface and the materials’ cytotoxicity. The biocompatibility of the samples after 120 °C heating was not tested, as in these samples toxic nitrate ions are present, as proved by FTIR analysis (Figure 1).

**Figure 4:**
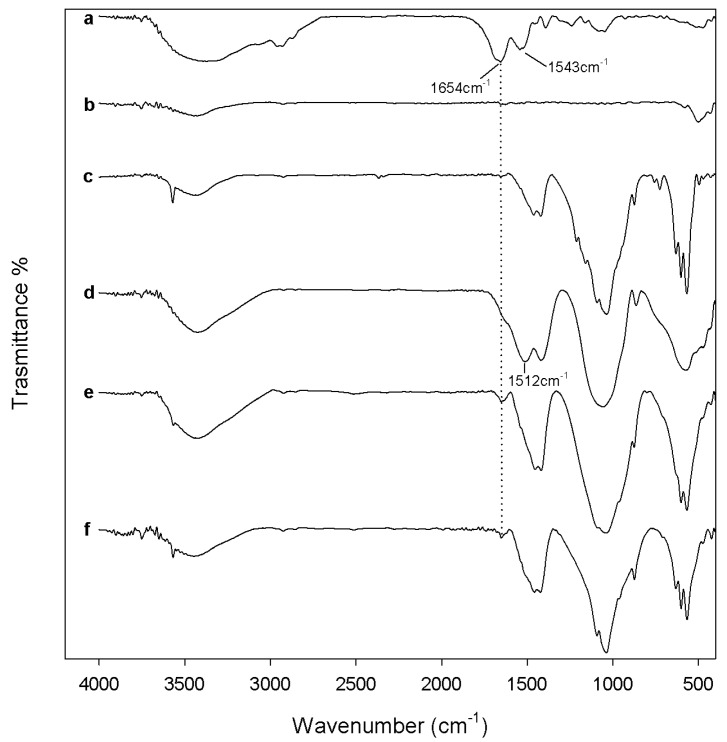
FTIR of (**a**) bovine serum albumin (BSA) and (**b**) ZrO_2_; (**c**) Hap; (**d**) 9ZrO_2_∙1Hap; (**e**) 7ZrO_2_∙3Hap; and (**f**) 5ZrO_2_∙5HAp gels, heated to 600 °C, after 24 h of exposure to the BSA solution.

**Figure 5:**
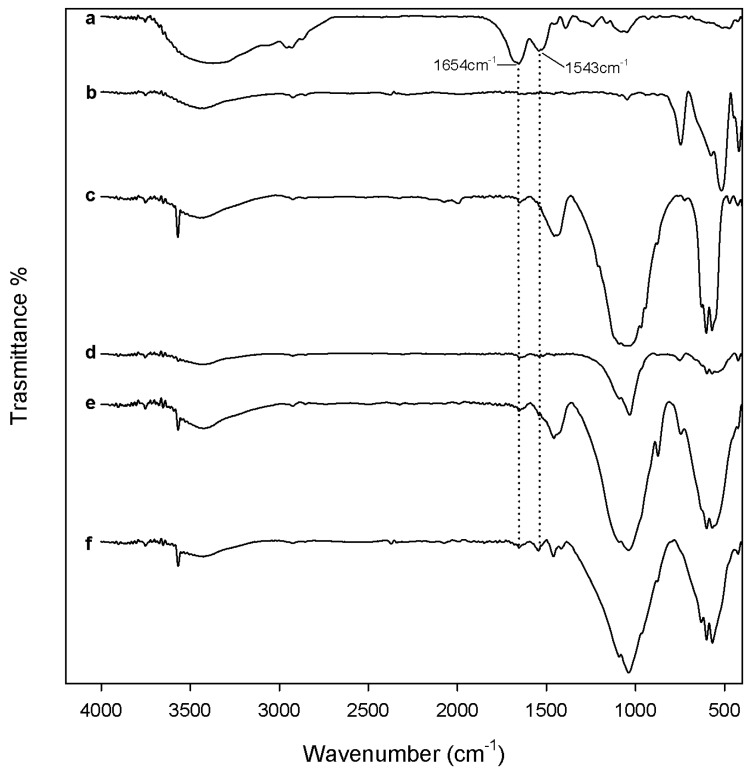
FTIR of (**a**) BSA and (**b**) ZrO_2_; (**c**) Hap; (**d**) 9ZrO_2_∙1Hap; (**e**) 7ZrO_2_∙3Hap; and (**f**) 5ZrO_2_∙5HAp gels, heated to 1000 °C, after 24 h of exposure to BSA solution.

Figure 4 and Figure 5 show FTIR spectra of all samples, heated to 600 °C and 1000 °C, respectively, after 24 h of exposure to bovine serum albumin (BSA) solution. The comparison between sample spectra (Figure 4, curves from b to e) and the BSA spectrum (Figure 4, curve a) showed that after heat treatment at 600 °C, the protein adsorption is low and occurs only on the surface of xZrO_2_·(1 − x)HAp composites. Indeed, the main peak of albumin at 1654 cm^−1^, due to the stretching of C-O in amide I [64], is visible with low intensity only in the FTIR spectra of the samples 0.9ZrO_2_·0.1HAp (as a shoulder of the band at 1512 cm^−1^), 0.7ZrO_2_·0.3Hap, and 0.5ZrO_2_·0.5HAp as weak peak.

The presence of the adsorbed proteins is more evident in the samples heat-treated to 1000 °C (Figure 5). In the spectra of all samples, except in the FTIR spectrum of pure ZrO_2_, an increase of the intensity of the albumin band at 1654 cm^−1^ is visible. Moreover, in the samples of 0.5ZrO_2_·0.5HAp the BSA band at 1543 cm^−1^ also appears, due to N–H in-plane bending of amide II [64]. Therefore, the protein adsorption is affected by heat treatment carried out on the materials after synthesis, and is higher when the materials are heated to 1000 °C. This can be ascribed to a different degree of ions’ release, from the materials heated to different temperatures. Mavropoulos et al. [65] proved that BSA adsorption on synthetic hydroxyapatite is affected by ions present in the solution containing the protein. In particular, the authors observed a decrease of the BSA adsorption with an increase of the phosphate concentration in the BSA solution. The presence of a high amount of PO_4_^3−^ in the diffusion layer at the HAp surface, indeed, results in an increase of the electrostatic repulsion force between HAp and BSA. Moreover, Catauro et al. [45] showed that calcium phosphates ternary systems, heated to 600 °C, release a higher amount of Ca^2+^ and PO_4_^3−^ ions compared to the same materials heated at 1000 °C. Therefore, the test results can be explained by a higher ion release from the materials heated to 600 °C, compared to those heated to 1000 °C (which modifies the pH of the solution, the charges of both the materials surface, and the BSA and, thus, affects their interaction). The higher ion release observed in the sol-gel materials heated to a lower temperature is due to their lower crystallinity degree, as reported in the literature [66].

Moreover, the presence of HAp improves the ZrO_2_ ability of absorbing proteins. As the blood protein adsorption on the sample surface is the first step leading to cell adhesion and proliferation, the test results suggest that the materials heated to 1000 °C are more biocompatible than those heated to 600 °C, regardless of the HA amount in the samples.

In order to confirm this data, WST-8 assay was carried out to evaluate material cytotoxicity.

**Figure 6:**
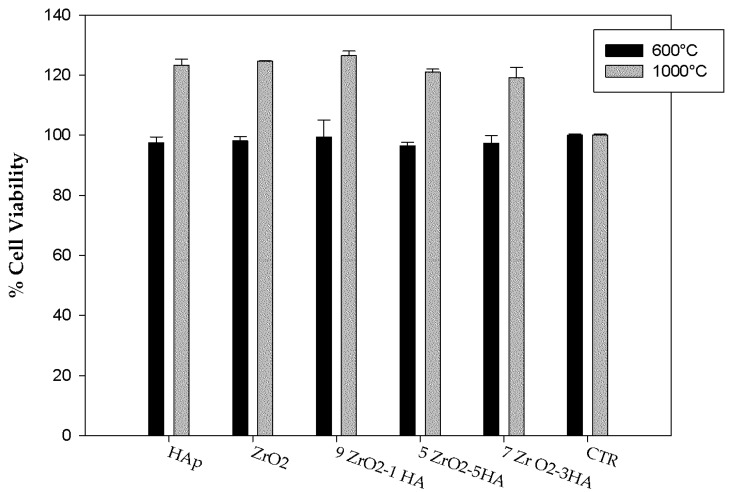
Cytotoxicity assay results. CTR: Control.

The results of the cytotoxicity assay are reported in Figure 6. NIH-3T3 (National Institutes of Health—3 day transfer, inoculum 3 × 10^5^ cells) murine fibroblast cell line, after contact with extracts of the samples heated to 600 °C, showed viability almost similar to control cells, regardless of the HAp amount. This result proves that both 600°-heated ZrO_2_ and Hap, as well as xZrO_2_·(1 − x)HAp composites, are bioinert materials. An increase of cell viability with respect to the control cells was recorded after exposure to extracts of the samples heated to 1000 °C.

Therefore, the HAp content does not affect the biocompatibility of the sol-gel materials, whereas the heat treatment influences this biological property. All samples (pure ZrO_2_ and Hap, as well as xZrO_2_·(1 − x)HAp composites), heated to 1000 °C, are more biocompatible than those heated to 600 °C, in agreement with the results of the protein adsorption test. This improvement can be due to the higher protein adsorption ability of the materials heated to 1000 °C. Moreover, as the biocompatibility improvement occurs also in pure materials (ZrO_2_ and HAp), it can be ascribed also to the zirconia and HAp microstructure modifications induced by heating. When the samples were heated to 1000 °C, an increase of crystallinity degree occurs and m-ZrO_2_ and TCP are formed (as proved by FTIR analysis, Figure 3), which can contribute to biocompatibility improvement of the samples. It is known, indeed, that t-ZrO_2_ is a bioinert material with good mechanical properties used in the dental field [1,3,5]. However, test results showed an increase of the viability of the cells seeded on m-ZrO_2_, suggesting that its presence makes the composites more biocompatible (Figure 6). Moreover, it is proven that TCP has higher osteoconductivity than HAp [67]. Therefore, its presence in the materials heated to 1000 °C, as well as the presence of m-ZrO_2_, can contribute to the improvement of the material’s biological performance.

The osseointegration ability of the sol-gel materials was evaluated by studying their bioactivity in vitro. After soaking in SBF and drying, powders and disks of the synthesized materials after 600 °C and 1000 °C heating were analyzed by FTIR and scanning electron microscopy (SEM).

**Figure 7:**
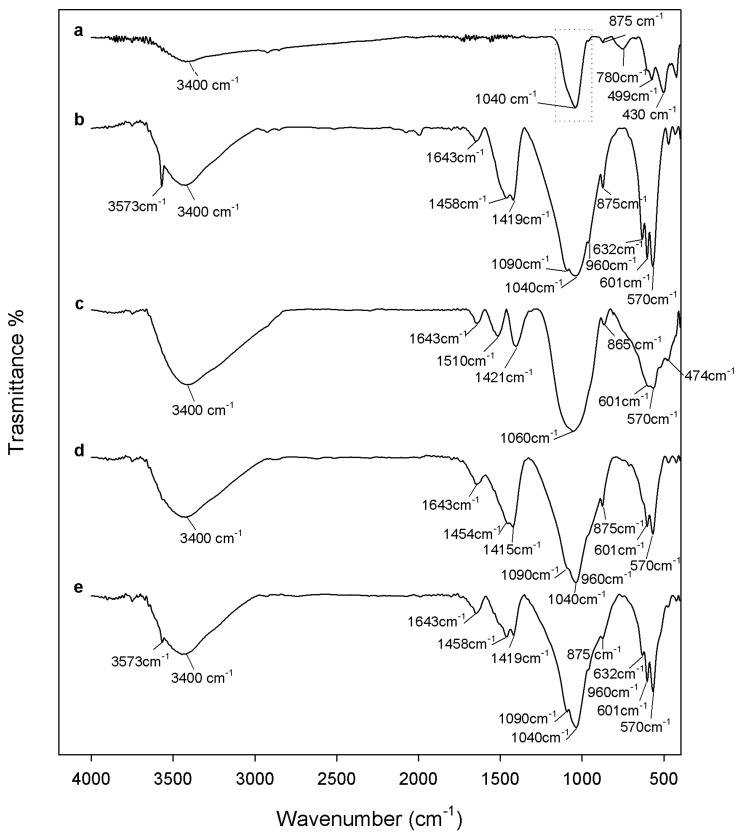
FTIR of (**a**) ZrO_2_; (**b**) Hap; (**c**) 9ZrO_2_∙1Hap; (**d**) 7ZrO_2_∙3Hap; and (**e**) 5ZrO_2_∙5HAp gels, heated to 600 °C, after 21 days of exposure to Simulated Body Fluid (SBF).

FTIR spectra of the pure HA and xZrO_2_·(1 − x)HAp samples heated to 600 °C, recorded after 21 days of exposure to SBF (Figure 7 curves from b to e), did not show any new peak compared to the FTIR spectra of the same samples recorded before the test (Figure 2). Only an increase and a broadening of the HAp signals are visible, ascribable to the nucleation of new HAp on the samples’ surfaces. 

In contrast, HAp nucleation on the pure ZrO_2_ sample (Figure 7 curve a) caused the appearance of a new intense peak at 1040 cm^−1^, due to P-O asymmetric stretching of the PO_4_^3−^ groups [54]. 

**Figure 8:**
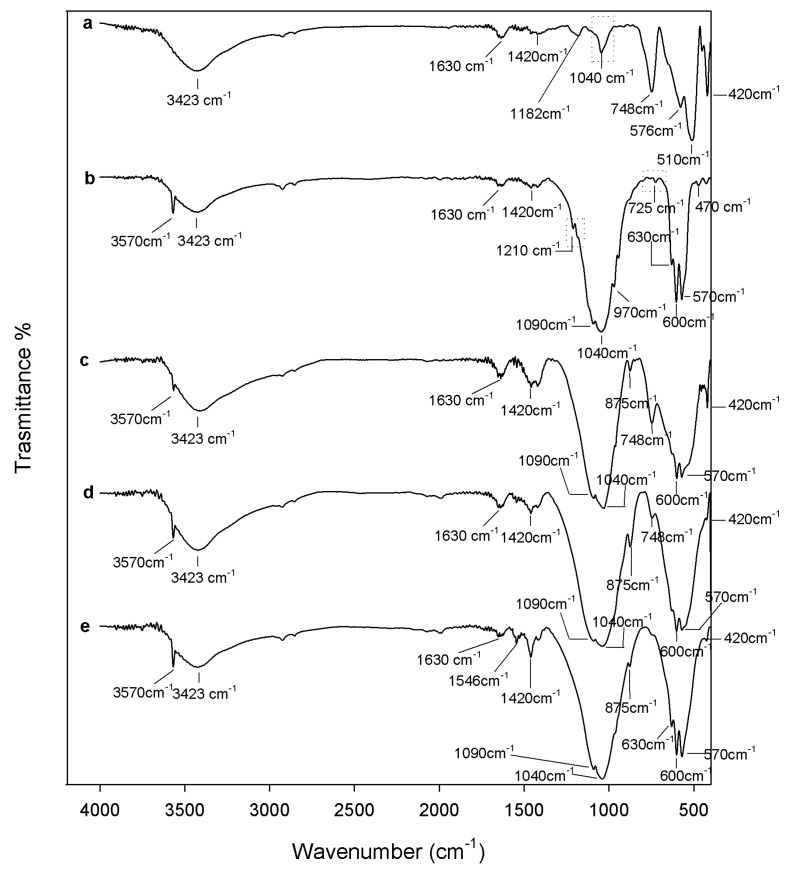
FTIR of (**a**) ZrO_2_; (**b**) HAp; (**c**) 9ZrO_2_∙1Hap; (**d**) 7ZrO_2_∙3Hap; and (**e**) 5ZrO_2_∙5HAp gels, heated to 1000 °C, after 21 days of exposure to SBF.

The change recorded in the spectra of the samples heated to 1000 °C after 21 days of exposure to SBF (Figure 8) are the same as those observed in the spectra of the samples heated to 600 °C. Therefore, a broadening and an intensity increase of the HAp signals is observable in all sample spectra and a peak at 1040 cm^−1^ [54], due to P-O asymmetric stretching in PO_4_^3−^ groups, is visible in the spectrum of the pure ZrO_2_ sample (Figure 8, curve a). Moreover, weak peaks at 1210 and 725 cm^−1^ are visible in the HAp spectrum (Figure 8, curve b), which can be assigned to P-O vibrations in pyrophosphate groups. The presence of pyrophosphate ion inclusions in the hydroxyapatite nucleated on the surface of the synthesized HAp can be due to the dissolution of the β-TCP (its formation was proved by FTIR analysis, Figure 3 curve b) in Simulated Body Fluid (SBF), which leads to the formation of HPO_4_^−2^, PO_4_^−3^, and OH^−^ ions [68]. As in the xZrO_2_·(1 − x)HAp composites, the amount of HAp is lower, while the content of β-TCP formed by HAp thermal decomposition is also lower than that present in HAp. Therefore, pyrophosphate signals are not visible in the composite spectra. 

The presence of the carbonate signals proves that calcium-deficient hydroxyapatite was grown on the surface of all synthesized samples. The carbonatation is higher in the 600 °C heated samples, i.e., their spectra carbonate signals are more evident. 

**Figure 9:**
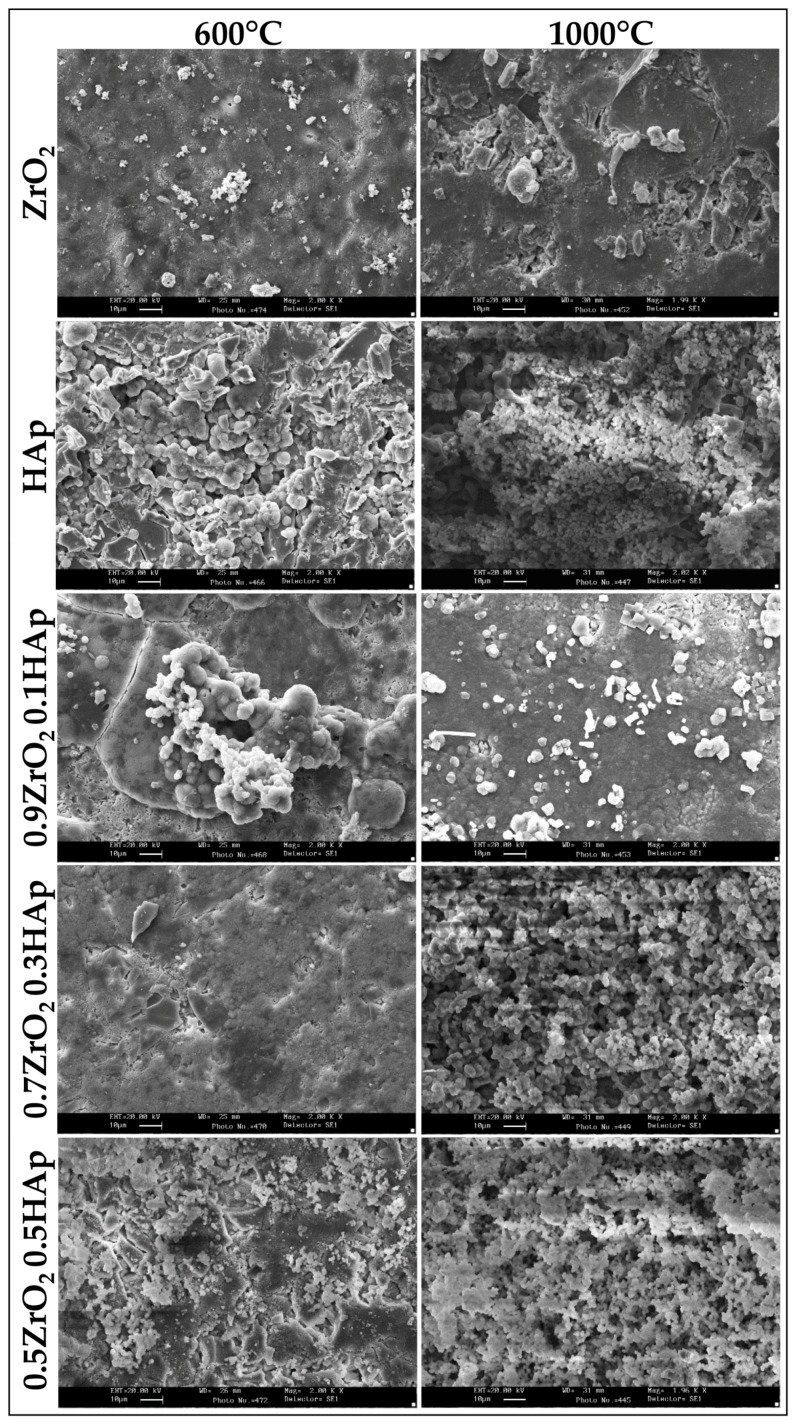
SEM images of all samples heated to 600 °C and 1000 °C after 21 days of exposure to SBF.

**Figure 10:**
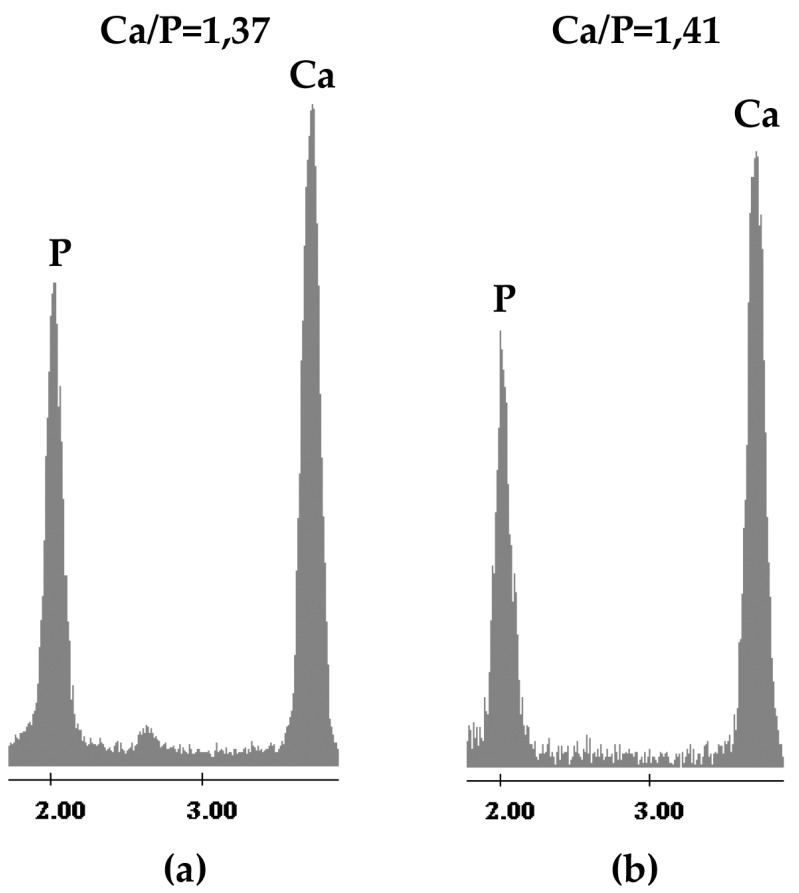
Representative Energy Dispersive X-ray analysis (EDX) images of the globular precipitate on the surface of the samples heated to (**a**) 600 °C and (**b**) 1000 °C after 21 days of exposure to SBF.

SEM/EDX (Energy Dispersive X-ray analysis) of the sample disks (Figure 9) confirmed FTIR results. On the surface of all samples the formation of a precipitate with a globular shape typical of HAp is visible (Figure 9). EDX analysis (Figure 10) of such globules show that they consist of an atomic ratio Ca/P < 1.67, identifying them as calcium-deficient HAp [69]. Therefore, all the synthesized materials are bioactive. However, on the surface of the 600°-heated samples only a few globules are visible, whereas the surface of the 1000°-heated samples, except to ZrO_2_, appears entirely covered by the HAp globule, confirming that 1000 °C heating improves the material’s bioactivity. The improvement of the bioactivity and the increase of the carbonatation process, recorded when the temperature of the heat treatment was increased from 600 °C to 1000 °C, can be ascribed to the different ion release degree caused by the different crystallinity degree of the materials. As already discussed above, in fact, the increase of crystallization, due to the 1000 °C heating, causes the decrease of the ion release in the SBF. The ion exchange, which occurs in the solution containing the 600 °C heated materials, is different from that which takes place in the solution containing the samples heated to 1000 °C. Therefore, the different ion exchange results in a different surface material charge and in a different pH of the SBF solution. The combination of those factors can modify the kinetic of both the nucleation reaction of HAp and of the CO_2_ solubilization process in SBF and, thus, of the carbonatation reaction. In the literature [45,70], the presence of a relationship between the heat treatment of the materials and the nucleation of the carbonated HAp was already observed and ascribed mainly to the increase of solution pH, due to the release of Ca^2+^ and PO_4_^3−^ ions [45].

Moreover, the SEM images showed that the pure HAp sample is more bioactive than pure ZrO_2_, and the higher the HAp content in the xZrO_2_·(1 − x)HAp composites, the higher the material’s bioactivity. This is ascribable mainly to the presence of Ca^2+^ ions. It is reported in the literature that a higher content of cation in the materials can induce HAp nucleation. The cations can be exchanged with the H^+^ in the SBF solution. The increase of the pH value of the solution leads to the dissociation of the –OH groups on the surface of the sol-gel materials and a higher amount of negatively charged –O^−^, able to induce HAp nucleation.

## 3. Materials and Methods 

### 3.1. Sol-Gel Synthesis of the Composites

The sol-gel method was used to synthesize xZrO_2_ (1 − x)HAp composites, where x is the mole fraction of ZrO_2_ in the composites and is equal to 1, 0.9, 0.7, 0.5, and 0. All reagents were obtained from Sigma Aldrich (Milan, Italy). To synthesize the pure ZrO_2_ sample (x = 1), a zirconium propoxide solution (70 wt. % in 1-propanol) was used as precursor. The metal alkoxide was added to a solution of acetyl acetone (AcAc) in ethanol 99.8%. The AcAc was added to act as inhibitor of the fast hydrolytic activity of zirconium propoxide. In the obtained sol the reagents had the following molar ratios: Zr(OCH_2_CH_2_CH_3_)_4_ /AcAc = 3 and EtOH/Zr(OCH_2_CH_2_CH_3_)_4_ = 6.

To synthesize the pure HAp sample (x = 0) two solutions were prepared, as follows:

1. Calcium nitrate tetrahydrate (Ca(NO_3_)_2_·4H_2_O) was dissolved in ethanol 99.8% under stirring;
2. Phosphorus pentoxide was added to a solution of NH_4_OH in Ethanol with pH = 11 under stirring.

When both the solutions were prepared, solution 1 was added to solution 2 under stirring. Within the obtained sol, the molar ratio Ca/P was equal to 1.67.

To synthesize the composites samples, the suitable amount of HAp sol (prepared as described above) was added drop by drop to ZrO_2_ sol under stirring. All obtained sols, homogeneous and transparent, were left to gel at room temperature. The gels were dried at 120 °C for 2 h and then heat treated at 600 °C and 1000 °C for 2 h.

**Figure 11:**
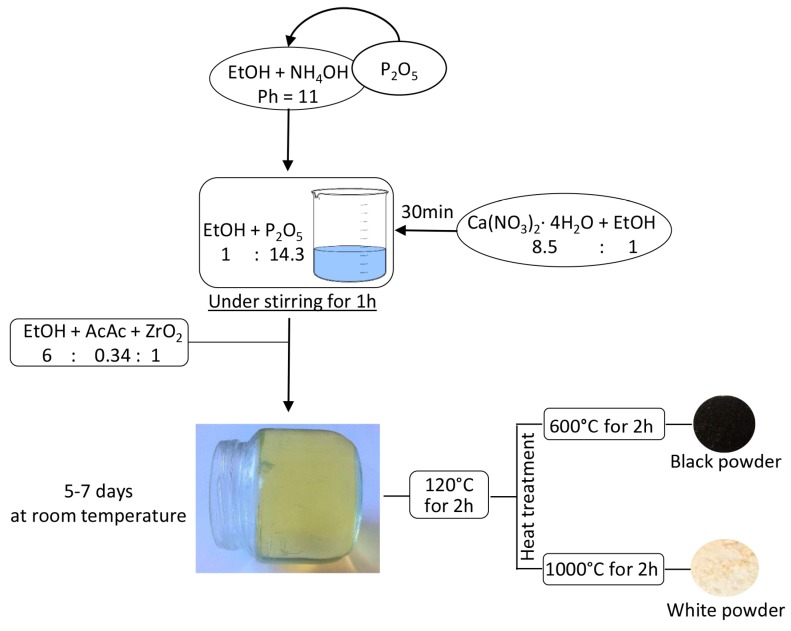
Flow chart of the sol-gel synthesis and heat treatments.

A schematic representation of the sol-gel synthesis is shown in Figure 11.

### 3.2. Composites Chemical Structure

The investigation of the chemical structure of the obtained composites was carried out on all the prepared samples after each heat treatment, in order to follow the evolution of the systems as a function of both the temperature and the relative amount of HAp in the composites.

Synthesized composites were analyzed using FTIR. Prestige 21 spectrophotometer (Shimadzu, Tokyo, Japan) was used to record transmittance spectra in the 400–4000 cm^−1^ region with a resolution of 4 cm^−1^ (45 scans). The instrument was equipped with a DTGS KBr (Deuterated Tryglycine Sulphate with potassium bromide windows) detector. Pelleted disks containing 2 mg of sample diluted with KBr (sample to KBr ratio = 1:100) were made. FTIR spectra were analyzed by Prestige software (IRsolution).

### 3.3. Biological Properties

#### 3.3.1. Protein Adsorption Evaluation

In order to evaluate the ability of the materials to interact with the blood proteins (the first step of the cell adhesion process), 10 mg/mL of each of the sample powders were soaked for 24 h in a solution of BSA, in a phosphate buffer with 2 mg/mL concentration. After soaking, the powders were gently rinsed three times with distilled water, and were dried at room temperature in a glass desiccator for 24 h. To evaluate the effective adsorption of the albumin on the materials, the dried powders were then analyzed by means of FTIR spectroscopy to assess the presence of the main peak of the albumin, at 1654 cm^−1^(stretching of C-O in amide I) and at 1543 cm^−1^ (N–H in-plane bending of amide II) [64]. 

#### 3.3.2. WST-8 Assay

Cytotoxicity evaluation was performed using the WST-8 assay (Dojindo Molecular Technologies Inc., MD, USA), a colorimetric assay. The extracted materials were obtained by incubating 150.0 mg of the sample powders for 2 h, in 7.5 mL of a complete culture medium, at 37 °C under continuous stirring. The NIH-3T3 murine fibroblast cell line (ATCC, Manassas VA, USA) was used in this cytotoxicity test. The cell line was grown in DMEM medium (Gibco, Gaithersburg, MD, USA) with 10% (*v*/*v*) fetal bovine serum (1% pen-strep) in a humidified incubator, at 37 °C and 5% CO_2_. Cells were seeded into 96 multiwell plates at a density of 5.0 × 10^3^ cells/well. After 24 h of incubation, cells were treated with the extracts for 24 h. Afterwards, they were washed 3 times with PBS (phosphate buffered saline) and again incubated with 10% *v*/*v* of WST-8 [2-(2-methoxy-4-nitrophenyl)-3-(4-nitrophenyl)-5-(2,4-disulfophenyl)-2H-tetrazolium, monosodium salt] in a fresh medium for 2 h. (the water-soluble purple-coloured WST-8 tetrazolium salt is able to penetrate the cellular membrane, and is cleaved by mitochondrial dehydrogenases of the live cells, producing insoluble yellow-orange crystals of formazan). The quantification of the generated formazan can be carried out measuring the absorbance, at 450 nm, of the well-plates, which is proportional to the number of viable cells. The absorbance value was measured with a UV-visible spectrophotometer (Biomate 3, Thermo Scientific, Walkersville, MD, USA). A low absorbance value means that the materials in contact with the cells are cytotoxic agents, able to inhibit their mitochondrial activity. Cell viability was expressed as a percentage of mitochondrial redox activity of the cells directly exposed to material extracts, compared to that of an unexposed control. Therefore, the value of the cell viability has been expressed as the percentage of UV absorbance at 450 nm, recorded in the well where the cells treated with the sample extracts were seeded (compared to the absorbance recorded in the well where untreated control cells where seeded, considered as 100% of viability). The percentages of cell viability were calculated as an average of 3 determinations ± the standard deviation.

#### 3.3.3. Apatite-Forming Ability Test

For evaluations of in vitro bioactivity, the apatite-forming ability test was carried out, as proposed by Kokubo et al. [19]. Both the sample powders and sample disks (13 mm of diameter and 2 mm of thickness), obtained by sample powder pressing, were soaked for 21 days in a SBF with an ion concentration nearly equal to that in the human blood plasma. Polystyrene bottles containing powder and SBF were placed in a water bath at 37.5 ± 0.5 °C. As the ratio between the exposed sample surface and the SBF volume affects the test, it was chosen for the powders and the disks in accordance with Catauro et al. [45] and Kokubo et al. [71], respectively, and kept constant. As the exposed surface area of the powders is higher than the one of the sample disks, a higher volume of SBS was used to test the bioactivity of the sample powders compared to the SBS volume used to test the sample disks.

The solution was replaced every 2 days to avoid depletion of the ionic species in the SBF, due to the nucleation of biominerals on the samples.

After each exposure time, the samples, as powders and as disks, were removed from SBF, gently washed with deionized water, and dried in a desiccator. The hydroxyapatite deposition on the powders was evaluated by FTIR spectroscopy, whereas on the disks it was evaluated by SEM(SEM Quanta 200, FEI, Eindhoven, The Netherlands), equipped with an EDX.

## 4. Conclusions

The sol-gel technique allowed the preparation of ZrO_2_-based composites containing different amounts of HAp. Modification of the materials’ structure was induced by heating (120 °C, 600 °C, and 1000 °C), and was followed by FTIR analysis of all samples after each heat treatment. Moreover, biological properties of the synthesized materials were tested in vitro as a function of heat treatment and HAp content. The results showed that HAp content does not influence the composites’ ability to adsorb blood proteins or their cytotoxicity, but improves the materials’ bioactivity. In contrast, the heat treatment acts on all the tested biological properties and, in particular, 1000 °C heating allowed a higher performance improvement than 600 °C heating. 

Therefore, the results of the reported preliminary tests encourage performing, in the future, more extensive analysis on cells attachment, proliferation, and viability. A more extensive biological characterization will be needed to fully understand the behavior of the synthesized materials in vitro, and to identify the cell-materials’ interaction mechanism and the effect of such interaction on the cell cycle.
